# *Cacna1b* alternative splicing impacts excitatory neurotransmission and is linked to behavioral responses to aversive stimuli

**DOI:** 10.1186/s13041-019-0500-1

**Published:** 2019-10-21

**Authors:** Alexandra Bunda, Brianna LaCarubba, Melanie Bertolino, Marie Akiki, Kevin Bath, Javier Lopez-Soto, Diane Lipscombe, Arturo Andrade

**Affiliations:** 1 Department of Biological Sciences, College of Life Sciences and Agriculture, University of New Hampshire, 46 College Road, Durham, NH 03824 USA; 2 Department of Cognitive, Linguistic and Psychological Sciences, Brown University, 190 Thayer Street, Providence, RI 02912 USA; 3 Robert J and Nancy D Carney Institute for Brain Science & Department of Neuroscience, Brown University, 185 Meeting Street, Providence, RI 02912 USA

**Keywords:** Calcium channels, Alternative splicing, Exploratory behavior, Anxiety, Medial entorhinal cortex, Dentate gyrus, Medial perforant path, Ca_V_2.2 channels

## Abstract

Presynaptic Ca_V_2.2 channels control calcium entry that triggers neurotransmitter release at both central and peripheral synapses. The *Cacna1b* gene encodes the α1-pore forming subunit of Ca_V_2.2 channels. Distinct subsets of splice variants of Ca_V_2.2 derived from cell-specific alternative splicing of the *Cacna1b* pre-mRNA are expressed in specific subpopulations of neurons. Four cell-specific sites of alternative splicing in *Cacna1b* that alter Ca_V_2.2 channel function have been described in detail: three cassette exons (e18a, e24a, and e31a) and a pair of mutually exclusive exons (e37a/e37b). *Cacna1b* mRNAs containing e37a are highly enriched in a subpopulation of nociceptors where they influence nociception and morphine analgesia. E37a-*Cacna1b* mRNAs are also expressed in brain, but their cell-specific expression in this part of the nervous system, their functional consequences in central synapses and their role on complex behavior have not been studied. In this report, we show that e37a-*Cacna1b* mRNAs are expressed in excitatory projection neurons where Ca_V_2.2 channels are known to influence transmitter release at excitatory inputs from entorhinal cortex (EC) to dentate gyrus (DG). By comparing behaviors of WT mice to those that only express e37b-Ca_V_2.2 channels, we found evidence that e37a-Ca_V_2.2 enhances behavioral responses to aversive stimuli. Our results suggest that alternative splicing of *Cacna1b* e37a influences excitatory transmitter release and couples to complex behaviors.

## Introduction

Presynaptic Ca_V_2.2 channels control neurotransmitter release throughout the nervous system where their activity impacts a wide range of neuronal functions. Ca_V_2.2 channels dominate in supporting transmission of sensory information from nociceptors to spinal cord dorsal horn neurons [1–6]. Mice that lack Ca_V_2.2 channels have impaired nociception [7–9] and intrathecal Ca_V_2.2 calcium channel blockers are analgesic [10]. Ca_V_2.2 channel activity also contributes to other behaviors in mice including locomotion, exploration, startle [9, 11], ethanol intake [12], and aggression [13]. In humans, inhibition of Ca_V_2.2 by intrathecal ziconotide can trigger psychotic episodes and anxiety, further emphasizing the importance of Ca_V_2.2 channels in higher level, brain function and complex behaviors [14–16].

The *Cacna1b* gene encodes the Ca_V_α_1_ pore-forming subunit of all Ca_V_2.2 channels, and alternatively spliced exons in *Cacna1b* influence channel function and sensitivity to G-protein coupled receptors (GPCRs) [17–20]. The expression of alternatively spliced exons in *Cacna1b* is regulated by factors that depend on tissue type, cell type, development, and disease state [21–24]. Of special interest are mutually exclusive exons, e37a and e37b, which encode sequences in the C-terminus of Ca_V_2.2 [25]. Previous work has shown that e37a-*Cacna1b* mRNAs are enriched in a subset of transient receptor potential vanilloid 1 (Trpv1) expressing nociceptors of dorsal root ganglia (DRG) [26]. Compared to e37b-Ca_V_2.2, e37a-Ca_V_2.2 channels are trafficked more efficiently to the cell surface [27, 28], they are inhibited more strongly by G_i/o_-protein coupled receptors including the μ-opioid receptor [19, 20, 29], and they enhance actions of intrathecal morphine analgesia in vivo [19, 20, 29]. E37a-Ca_V_2.2 and e37b-Ca_V_2.2 channels contribute to basal thermal and mechanical nociception but e37a-Ca_V_2.2 channels have a preferred role in the maintenance of thermal and mechanical hyperalgesia induced by inflammation [30, 31]. The unique function of e37a-Ca_V_2.2 channels in nociception and sensitivity to GPCR inhibition motivated us to explore the expression pattern and potential function of e37a-Ca_V_2.2 channels in brain.

Here we show that e37a-*Cacna1b* mRNAs are expressed at low level throughout the brain but they are enriched in subsets of excitatory projection neurons. E37a-*Cacna1b* mRNAs are more abundant in Ca^2+^/calmodulin-dependent protein kinase II excitatory projection neurons (CaMKIIα^+^PNs) compared to cholecystokinin-expressing interneurons (CCK^+^INs). We show that e37a-Ca_V_2.2 channels contribute to transmitter release at cortico-hippocampal excitatory synapses and they inhibit exploratory and novelty-induced anxiety-like behaviors. Our results suggest that e37a-Ca_V_2.2 channels influence presynaptic transmitter release at specific synapses in the brain and are linked to behavioral responses to aversive stimuli.

## Methods

### Transgenic mice

All of experimental procedures followed the guidelines of the Institutional Animal Care and Use Committee of Brown University and the University of New Hampshire. Adult male and female mice were used in all experiments. Mice were housed with food and water ad libitum in temperature-controlled rooms with a 12 h light/dark cycle. E37b-only mice (*Cacna1b*^*tm2.1Dili*^) were backcrossed for at least six generations in C57BL6 (Charles River) and were generated as described previously [19, 31]. C57BL/6 wild-type (WT) mice were used as controls. For experiments using mice with a C57BL/6;I129 mixed background, WT mice were bred in parallel with e37b-only mice to obtain matched genetic backgrounds. TdTomato (tdT) was expressed in CCK^+^INs by intersectional genetic labeling as reported [32, 33]. Briefly, we crossed *CCK-ires-cre* (*Cck*^*tm1.1(cre)Zjh*^/J, Jax:012706) to *Dlx5/6-Flpe* (*Tg (mI56i-flpe)39Fsh/J*, Jax: 010815) twice to generate dual transgenic mice (homozygous for *CCK-ires-cre* and heterozygous for *Dlx5/6-Flpe*), then crossed these strain to *RCFL-tdT* strain (*B6;129S-Gt (ROSA)26Sort*^*m65.1(CAG-tdTomato)Hze*^*/J*, Jax: 021875) to generate mice heterozygous for three alleles (*CCK-ires-Cre::Dlx5/6-Flpe::RCFL-tdT*). This strain will be referred to as *CCK;Dlx5/6;tdT*. Mouse strains expressing tdT in CaMKIIα^+^PNs were generated by crossing *CaMKIIα-Cre* mice (*B6.Cg-Tg (Camk2a-cre)T29-1Stl/J*, Jax: 005359) to *Ai14* mice (*B6.Cg-Gt (ROSA)26Sor*^*tm14(CAG-tdTomato)Hze*^*/J*, Jax: 007914). The dual transgenic mouse strain *CaMKIIα::Ai14* was heterozygous for both alleles, this strain will be referred to as *CaMKIIα;tdT*.

### Genotyping

Genomic DNA was extracted from P7-P9 animal tissue using Phire Animal Tissue Direct kit II (ThermoFisher Scientific, F140WH) according to manufacturer instructions. PCR was performed with AmpliTaq Gold® 360 mastermix (Thermo Fisher Scientific, 4,398,881) using the following conditions: a hot start of 95^0^ C for 10 min, followed by 35 cycles (95^0^ C, 30 s; 60^0^ C, 30 s; 72^0^ C, 1 min), and a final step of 72^0^ C for 7 min. Primers and expected products are shown in Table 1. Primers were added to the same mixture for genotyping.

**Table 1:** Primers and expected products

Mouse line	Primers	Expected products
*CCK-Cre*	F-WT1: GGGAGGCAGATAGGATCACA F-MT1: TGGTTTGTCCAAACTCATCAA R: GAGGGGTCGTATGTGTGGTT	Hom: 180 bp Het: 180 bp and 468 bp WT: 468 bp
*Dlx5/6-Flpe*	F-T1: CAGAATTGATCCTGGGGAGCTACG R-T1: CCAGGACCTTAGGTGGTGTTTTAC F-C: CAAATGTTGCTTGTCTGGTG R-C: GTCAGTCGAGTGCACAGT TT	Transgene: 406 bp PCR positive control: 200 bp *PCR conditions do not differentiate between heterozygous and homozygous mice
*Ai14 and RCFL-tdT*	F-WT2: AAGGGAGCTGCAGTGGAG TA R-WT2: CCGAAAATCTGTGGGAAG TC R-MT1: GGCATTAAAGCAGCGTAT CC F-MT2: CTGTTCCTGTACGGCATGG	Hom: 196 Het: 196 and 297 WT: 297
*CaMKIIα-Cre*	F-T1: GTT CTC CGT TTG CAC TCA GG R-T1: CAG GTT CTT GCG AAC CTC AT F-C: AGT GGC CTC TTC CAG AAA TG R-C: TGC GAC TGT GTC TGA TTT CC	Transgene: ~ 500 bp PCR positive control: 521 bp

All primers are reported in 5′ to 3′ direction

### Microdissections and RT-PCR

C57BL/6 male adults were deeply anesthetized with isofluorane; 400 μm brain slices were prepared in ice-cold artificial cerebrospinal fluid (ACSF) in mM: NaCl 130, KCl 3.5, KH_2_PO_4_ 1.1, MgCl_2_ 6.0, CaCl_2_ 1.0, dextrose 10, kynurenate 2.0, NaHCO_3_ 30, ascorbate 0.4, thiourea 0.8, Na-Pyruvate 2.0 using a Leica VT 1000S vibratome (Leica Microsystems). Medial prefrontal cortex (mPFC), amygdala (AMY), and ventral hippocampus (vHPC) were microdissected under constant oxygenation. 3–6 dissected brain regions from at least three different mice were pooled, tissue was flash frozen with dry ice and stored at − 80 °C. Next, tissue was mechanically homogenized using TRIzol® (ThermoFisher, 15,596,026). After adding chloroform and centrifuging, RNA was extracted RNeasy® Mini Kit columns (Qiagen, 74,134). 200 ng of total RNA was used to produce cDNA with SuperScript™ III First-Strand system (ThermoFisher Scientific, 18,080–051). PCR was performed with AmpliTaq Gold® 360 (ThermoFisher Scientific, 4,398,886) using F-37a: 5′-AGGCCTGGCATGAGATCATGC and R-37a: 5′-CCTACGAGGGCAGTTCTTTCC primers and the following conditions: a hot start at 95 °C for 10 min, 35 cycles (95 °C for 30 s, 60 °C for 30 s, 72 °C for 1 min), and a final step of 72 °C for 7 min. To confirm amplicon identity, PCR products were digested with BsrGI (New England Biolabs, R0575S) overnight at 37 °C. Digested PCR products were run in 3% agarose and stained with ethidium bromide. Plasmids containing cDNA for 37a-Ca_V_2.2 were used as positive control (Addgene, plasmid #26569).

### In-situ hybridization (BaseScope™)

To detect e37a-*Cacna1b* splice variants in brain tissue, we utilized a variation of in-situ hybridization, BaseScope™. Briefly, ‘Z’ probes contained a complementary sequence that binds *Cacna1b* mRNA between e36 and e37a, and a signal amplification complex containing the Fast Red dye. The punctate pattern of the Z probe signals is due to the ability of this technique to detect single RNA molecules [34]. Hematoxylin counterstaining was used to label nuclei. In brief, deeply anesthetized adult C57BL/6 mice either males or females (Euthasol, Virbac, 200–071) were transcardially perfused with 1x PBS and subsequently with 10% neutral buffered formalin (NBF) fixative solution (Sigma, HT501128). Brains were post-fixated in 10% NBF at 4 °C for 24 h, washed 1x PBS, and dehydrated in a PBS-15% sucrose solution for at least 18 h at 4 °C, or until tissue sank to the bottom of the tube. Tissue was transferred into PBS-30% sucrose solution for a second dehydration step for about 18 h at 4 °C or until tissue sank to the bottom of the tube. Next, tissue was cryopreserved in optimal cutting temperature formulation (Fisher, 4585), with isopentane pre-chilled in dry ice. 12 μm sections from brain were generated in a cryotome (Shandon, 77,200,222) and individual sections were placed in 15 mm Netwell™ inserts (Corning, 3478). Sections were allowed to free-float in 1x PBS, and mounted on positively charged microscope slides (VWR, 48311–703). Sections were air-dried 10–20 min before in-situ hybridization (ISH). To aid tissue adhesion, air-dried sections were incubated at 60 °C in a drying oven for 30 min, then post-fixed in 10% NBF at 4 °C for 15 min and immediately transferred to 50, 70%, and two rounds of 100% ethanol for 5 min each. Slides were then allowed to air dry for additional 5 min before incubating with RNAscope® hydrogen peroxide solution for 10 min (ACD, 322381). Sections were then washed in Milli-Q® water and transferred to RNAscope® Target Retrieval solution preheated to 99 °C for 15 min (ACD, 322000). Sections were briefly washed in Milli-Q® water, transferred to 100% ethanol for 3 min, then placed in drying oven at 60 °C for 30 min. Sections were isolated with a hydrophobic barrier pen (ACD, 310018) and allowed to air-dry overnight at RT. Next sections were incubated with RNAscope® Protease III for 30 min (ACD, 322381) at 40 °C in the ACD HybEZ™ Hybridization System (ACD, 310010). BaseScope™ probe spanning exon junction 36:37a of *Cacna1b* mRNA (ACD, 701151) was applied to each section for 2 h at 40 °C in the ACD HybEZ™ Hybridization System. BaseScope™ Detection Reagents AMP 0 - AMP 6 and FastRed (ACD, 322910) were applied according to manufacturer protocol and washed using RNAscope® Wash Buffer (ACD, 310091). To visualize nuclei, sections were counterstained in Gil’s Hematoxylin I for 2 min at RT (American Master Tech, HXGHE) following signal amplification. Sections were washed in tap water 3 times, briefly transferred to 0.02%ammonia water, and washed again in tap water. Finally, sections were dried at 60 °C for 15 min and mounted using VectaMount™ mounting medium (Vector Laboratories, H-5000). Cell counting was performed using Fiji cell counter plug in [35, 36].

**Fig. 1:**
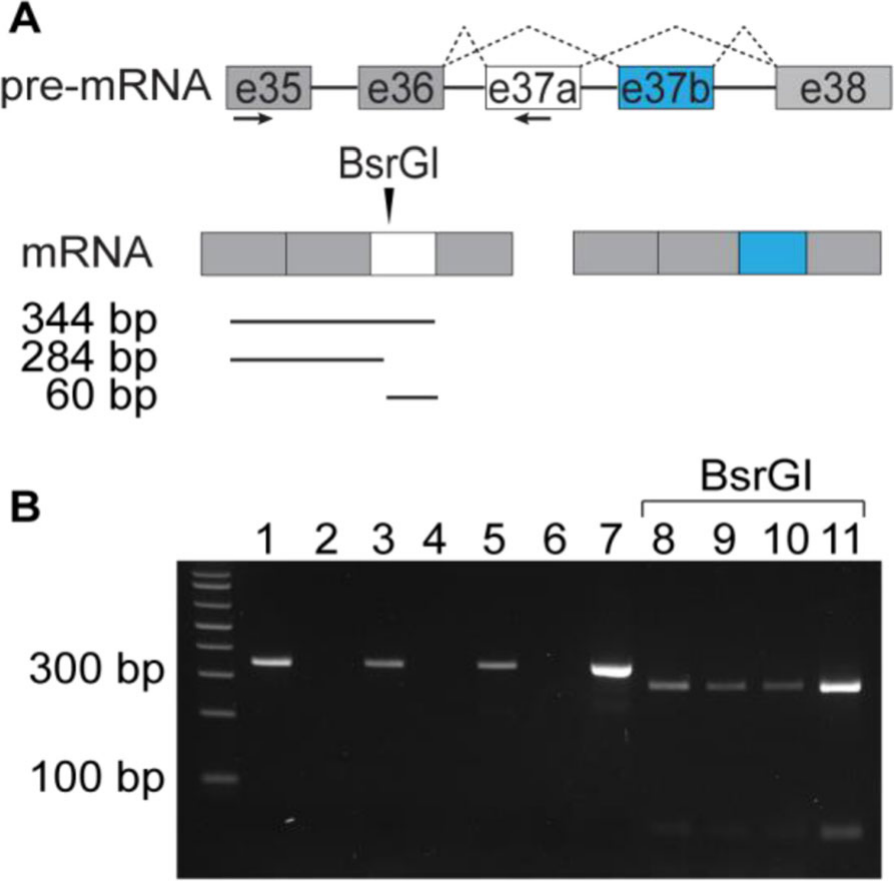
e37a-*Cacna1b* splice isoforms are expressed in mPFC, vHPC and AMY. **a** Schematic showing the approximate location of PCR primers to amplify e37a-containing cDNA (horizontal arrows), the approximate location of a unique BsrGI site within the e37a sequence (vertical arrow), and the expected size for BsrGI digested products. **b** Representative image of an agarose gel electrophoresis of undigested PCR products of RT samples from mPFC, vHPC and AMY (lanes 1, 3, and 5 respectively). Negative controls for each RNA sample without reverse transcriptase (lanes 2, 4, and 6 respectively). Positive control with a plasmid that contains cDNA for e37a-*Cacna1b* (lane 7). BsrGI digested PCR products derived from RT-PCRs from mPFC, vHPC, AMY, and e37a-*Cacna1b* plasmid (lanes 8, 9, 10, 11 respectively)

### Fluorescence-activated cell sorting (FACS)

Adult *CCK;Dlx5/6;tdT* and *CaMKIIα;tdT* mice were deeply anesthetized with isofluorane, brains removed and dissected (in less than 45 s) in Earl’s Balanced Salt Solution (EBSS) (Sigma, E3024) containing 21 U/mL of papain. Cerebral cortex or hippocampal tissue was dissociated using a modified version of Worthington Papain Dissociating System® (Worthington Biochemical Corporation, LK003150). After incubating with papain for 45 min at 37 °C on a rocking platform, tissue was triturated with three sequential diameter fire-polished glass pipettes. Next, cell suspensions were centrifuged at 300 x g for 5 min. After discarding supernatants, pellets were resuspended in 3 mL of EBSS containing 0.1% of ovomucoid protease inhibitor and 0.1% bovine serum albumin (Worthington, LK003182) to quench papain. Cell suspension was centrifuged at 270 x g for 6 min and resuspended in EBSS (3 mL). To isolate tdT-expressing cells, we performed FACS in a Sony SH800 flow cytometer using a 561-nm laser to excite and a 570–630 nm filter for event selection. At least 300,000 events were collected directly into TRIzol™ LS Reagent (ThermoFisher Scientific, 10,296,028). Collection was performed keeping 1:3 (v/v) sorted cell suspension: TRIzol™ LS ratio. Cell suspension were kept on ice throughout the sorting session.

### RT-qPCR

Total RNA from sorted cells was extracted using TRIzol™ LS and isopropanol precipitation with the addition of 30 μg of GlycoBlue® Coprecipitant (ThermoFisher Scientific, AM9516) to facilitate visualization of RNA pellet. 300 ng of total RNA from sorted cells was primed with oligo-dT and reverse transcribed with Superscript IV First-Strand Synthesis System (ThermoFisher Scientific, 18,091,050) according to manufacturer instructions. To quantify the amounts of 37a relative to total Ca_V_2.2 mRNA, we used a set of primers that amplify e37a. A forward primer was designed to target the splice junction between e36 and e37a (e36-37a: CTGCGTG.

TTGCCGGATT) and a reverse primer to target a sequence within e37a (e36-37aR: 5’ACCTACGAGGGCAGTTCTT). The e36-37a amplification was normalized to a PCR reaction using a set of primers that amplify between constitutive exons 35 and 36 (e35–36F: 5′ GGAAACATTGCCCTTGATGATG, e35–36R 5′ CAGTGGCACTCCTGAACAATA. Fig. 6c). The amplification efficiencies of both sets of primers were tested using serial dilutions of cDNA obtained from DRG samples (Fig. 6d). First-strand cDNA was diluted 1:5 and 4 μL of this dilution were used in a 20 μL qPCR reaction containing 10 μL of EvaGreen® 2X mastermix (Biotum, 31,003) and 0.8 μL of forward and reverse primers (10 μM). RT-qPCR reactions were run on an ABI 7500 Fast Real-Time PCR system (Applied Biosystems) with the following conditions: 1 cycle 95 °C for 2 min, 45 cycles (95 °C for 15 s and 60 °C for 1 min). Each sample from at least five different mice per genotype (biological replicates) was run in triplicate (technical replicates). Ct values were determined by 7500 Software v2.3 (Applied Biosystems). Relative quantification of gene expression was performed with the 2^-ΔΔCt^ method [37]. To confirm band identity, all bands were cloned and sequenced using NEB® PCR Cloning Kit (NEB, E1202S). End-point PCR was performed to validate the specificity of primers directed to 37a (Fig. 6c). We used 37a-*Cacna1b* cDNA clone (Addgene, plasmid #26569) and 37b-*Cacna1b* cDNA clone (Addgene, plasmid #26571). Glutamate decarboxylase 2 (*Gad2*) mRNA was quantified using TaqMan® real-time PCR assays (ThermoFisher Scientific) with probe Mm00484623_m1. Levels of mRNA were normalized to glyceraldehyde 3-phosphate dehydrogenase (*Gapdh*) using the probe Mm99999915_g1. Cycling conditions were similar to those described above.

### Behavioral assays

#### Elevated plus maze

After 30 min of habituation to the testing room, mice were placed on the elevated plus maze. Mice were recorded for 10 min with an infrared-sensitive digital camera in dimmed light. After each trial, the maze was cleaned with clorhexidine gluconate to reduce odor cues by previous subjects. The time spent in open areas and the frequency of boundary crosses were recorded and analyzed with Ethovision XT 8.0 (Noldus, Leesburg, VA). Exploratory behavior was measured by percent time spent in and percent of entries into the open arms.

#### Novelty induced Hypophagia

Prior to testing, mice were housed 2–3 per cage. Mice received 3 consecutive days of training (days 1–3) in a dark room in their home cage to find a reward (sweet milk). Training sessions consisted of presenting mice with a standard dual bearing sipper tube (Quick Quench 5 oz. bottle) inserted between the wire bars of the cage roof and containing 1:3 sweetened condensed milk with water. Mice were trained to find the sweet milk in their home cage (HC) in the dark for 3 days. On the fourth day mice were tested in their HC in the dark room. For testing, all but one of the mice were removed from their HC and placed into a holding cage containing shavings taken from its own HC. The mouse left in the HC was exposed to the sweet milk solution. This indicates the beginning of the trial. The latency to drink was recorded over a 5-min period with an infrared camera. Following completion of the 5-min trial, the second and third (if applicable) animals were rotated into the HC and tested in the same manner. On fifth day, novel cage (NC) testing was conducted by placing a single mouse into a clean cage of the same dimensions as their HC, but with no shavings and under bright lighting conditions. Mice were again presented with a sipper tube bottle of diluted sweetened milk, and the latency to drink was determined off-line using a digital timer. Novelty induced anxiety-like behavior was measured by the relative difference between HC and NC in the latency to approach reward.

### Brain slice electrophysiology

Briefly, adult mice were deeply anesthetized with isofluorane. The brain was immediately removed and rapidly placed in ice-cold artificial cerebrospinal fluid ACSF (mM): NaCl, 119; NaHCO_3_, 26; KCl, 2.5; NaH_2_PO_4_, 1; CaCl_2_, 2.5; MgSO_4_, 1.3; Dextrose; 11.0. 400 μm coronal slices from vHPC were prepared using a Leica 1000 vibrating slicer. After cutting, slices were held 1 h at room temperature in ACSF with constant oxygenation, then transferred to the recording chamber with continuous perfusion (1–2 ml/min). Field excitatory postsynaptic potentials (fEPSPs) were recorded by placing a microelectrode filled with ACSF into the superior blade of DG. A bipolar tungsten stimulation electrode (FHC Inc) was placed in nearby in this layer of DG and the medial perforant path (mPP) was identified by pre-pulse inhibition. Stimuli were applied using a current stimulus isolator (A365, WPI Inc). To ensure consistency in current application the output current from the stimulus unit was measured before the experiment was performed. Recordings were filtered at 2 kHz and digitalized at 20 kHz using a multiclamp 700A amplifier and acquired with Clampex 10.2 (Molecular Devices). The initial fEPSP slope was measured by fitting a straight line using Clampfit 10.2 (Molecular Devices). At least 2 slices were recorded per mouse per genotype.

### Statistical analysis

Student’s t-test and ANOVA repeated measures were performed using SPSS Statistics (IBM). The genotype was unknown to the experimenter during data acquisition and during analysis for all experiments.

## Results

### E37a-*Cacna1b* mRNAs are expressed in brain

We were interested in knowing if e37a-*Cacna1b* mRNAs were expressed in discrete regions of the brain. We used exon-specific primers for RT-PCR to test if e37a-*Cacna1b* mRNAs are expressed in medial prefrontal cortex (mPFC), ventral hippocampus (vHPC), and amygdala (AMY). We RT-PCR amplified RNA from micro-dissected tissue using primers in constitutive exon 35 and alternative exon 37a (Fig. 1a). PCR products of the expected size (~ 340 bp) were amplified from all three brain regions (Fig. 1b, *lanes 1, 3, 5*). BsrGI digest of PCR amplified products generated two bands of the predicted size for e37a (~ 280 and ~ 60 bp) for all three brain regions and 37a-Ca_V_2.2 cDNA control (Fig. 1a, *lanes 8, 9, 10 and 11, respectively*).

**Fig. 2:**
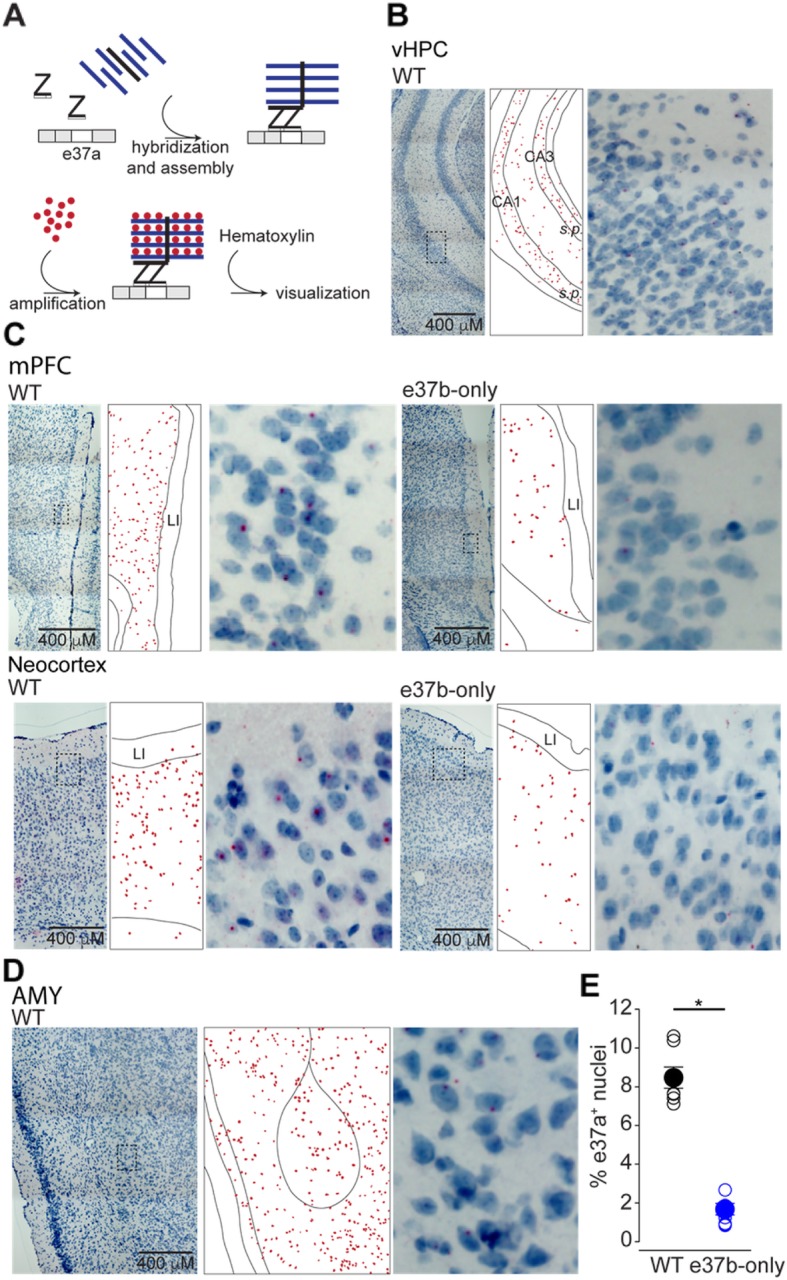
Localization of e37a-*Cacna1b* mRNA in vHPC, mPFC, neocortex and AMY. **a** Schematic depicting BaseScope™ workflow used to detect e37a-*Cacna1b* mRNA in brain sections. **b** Representative BaseScope™ images of vHPC from sections of WT mice. **c** mPFC section from WT and e37b-only mice, *left* and *right* respectively. **d** *Bottom left* and *right* show representative images from AMY of sections from WT mice. In **b-d**, *middle panels* indicate localization of nuclei stained with e37a (red dots). These red dots were constructed relative to the image shown in the corresponding *left panel. Right panel* shows amplified insets from *left panel*. **e** Comparisons of nuclei containing signal for e37a (e37a^+^) between WT and e37b-only mice. Percent e37a^+^ nuclei was calculated by normalizing the number e37a^+^ nuclei to all nuclei stained with hematoxylin in cortical sections of mice from both genotypes. Data are represented as mean (filled symbols) ± SE and individual values (empty symbols). * *p* < 0.05

To obtain more information about the cell-specific expression pattern of e37a-*Cacna1b* mRNA we used BaseScope™ in situ hybridization. The specificity of e37a-specific probes was confirmed using brain sections of mice null for e37a (e37b-only mice) [19]. In WT mice, e37a-signal was observed in cell nuclei likely because transcription and mRNA splicing are coupled [38]. In the vHPC, the e37a signal was observed in nuclei of CA1 and CA3 pyramidal layers, *stratum pyramidale (s.p.*), as well as in interneuron populated layers, *stratum radiatum (s.r.)*, *lacunosum-moleculare (s.l.m.), stratum lucidum (s.l.), and stratum oriens (s.o.)* (Fig. 2b). mPFC and neocortex contained e37a-*Cacna1b* mRNA puncta in nuclei, but the e37a-*Cacna1b* expression pattern was distributed through all cortical layers except for layer I (LI), and not restricted to any specific cell layer (Fig. 2c). E37a-containing nuclei were scattered throughout AMY as well (Fig. 2d). To assess the specificity of the e37a probe, we compared the percentage of nuclei showing signal for e37a in sections from WT and e37b-only mice. We found that ~ 8.5% of nuclei were positive for e37a in sections from WT mice, compared to ~ 1.6% in e37b-only mice (% e37a^+^ nuclei ± SE. WT = 8.5 ± 0.55, *n* = 6; e37b-only = 1.7 ± 0.30, *n* = 4. Mann-Whitney test, *p* < 0.003, Fig. 2e). These results indicate the majority of the signal is specific for e37a-*Cacna1b* mRNA, although ~ 20% of the signal is non-specific.

**Fig. 3:**
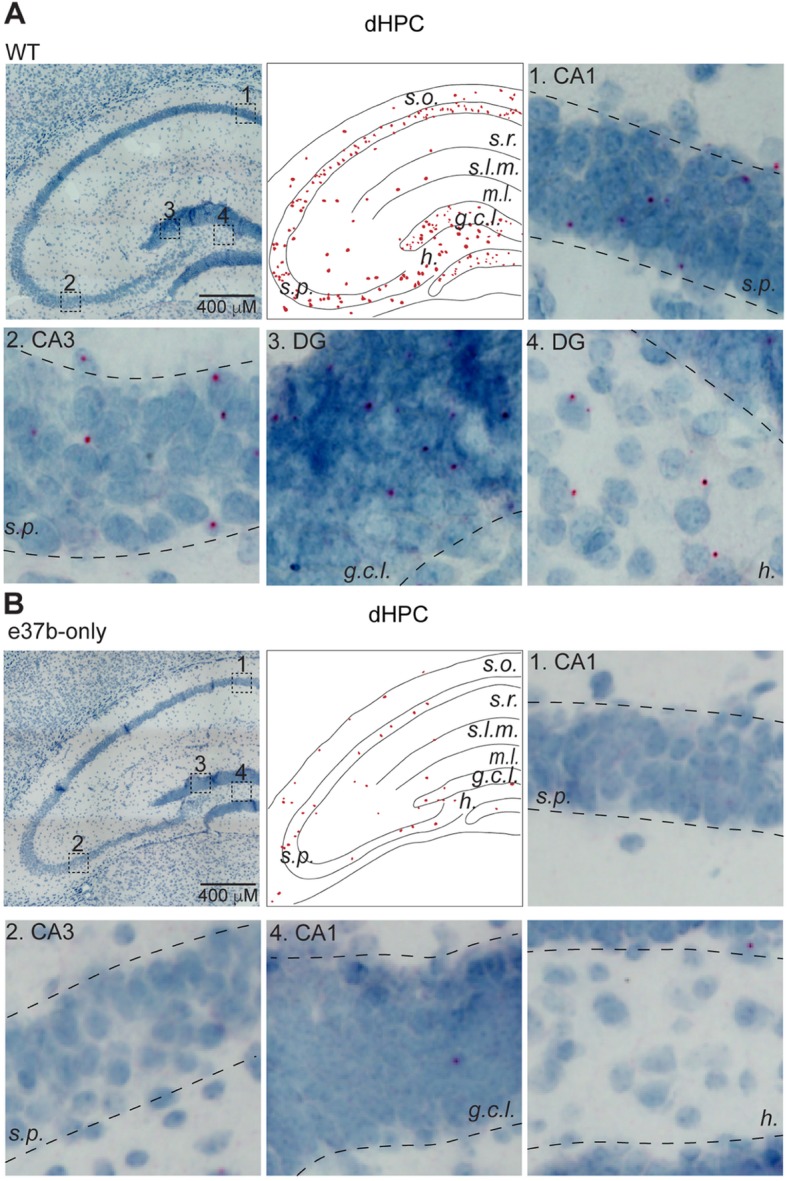
Distribution of e37a-*Cacna1b* mRNA in dHPC. **a** and **b** Representative BaseScope™ images of dorsal hippocampus (dHPC) sections from WT mice (**a**). *Middle upper panel* shows the localization of e37a^+^ nuclei within the dHPC in sections of WT (**a**) and e37b-only (**b**) mice. These images were constructed from the corresponding *right upper panel* image. Insets 1–4 were amplified for clarity to show regions of *s. p.*, *s. r., g.c.l. and h.* in sections from WT (**a**) and e37b-only mice (**b**)

Similar to the vHPC, we observed an enrichment of e37a-*Cacna1b* in dorsal hippocampus (dHPC) in *s.p., s.r.*, *s.l.m.* and *s.o.* layers (Fig. 3a). In the DG, e37a signal was observed in the granular cell layer (*g.c.l.*) and hilus (*h.*) but not in the molecular layer (*m.l.*) (Fig. 3a). Some background e37a signal was observed in similar brain areas in e37b-only mice, however this represented only ~ 20% relative to brain sections from WT mice (Fig. 3b). Our results show that e37a-*Cacna1b* mRNA is expressed in cortical and hippocampal areas including in projection neurons in *s.p*. Given the presence of projection neurons in *s.p.,* we tested if e37a-Ca_V_2.2 channels contribute to synaptic responses at glutamatergic synapses.

### 37a-Ca_V_2.2 channels influence release probability at medial perforant path-dentate gyrus synapses

**Fig. 4:**
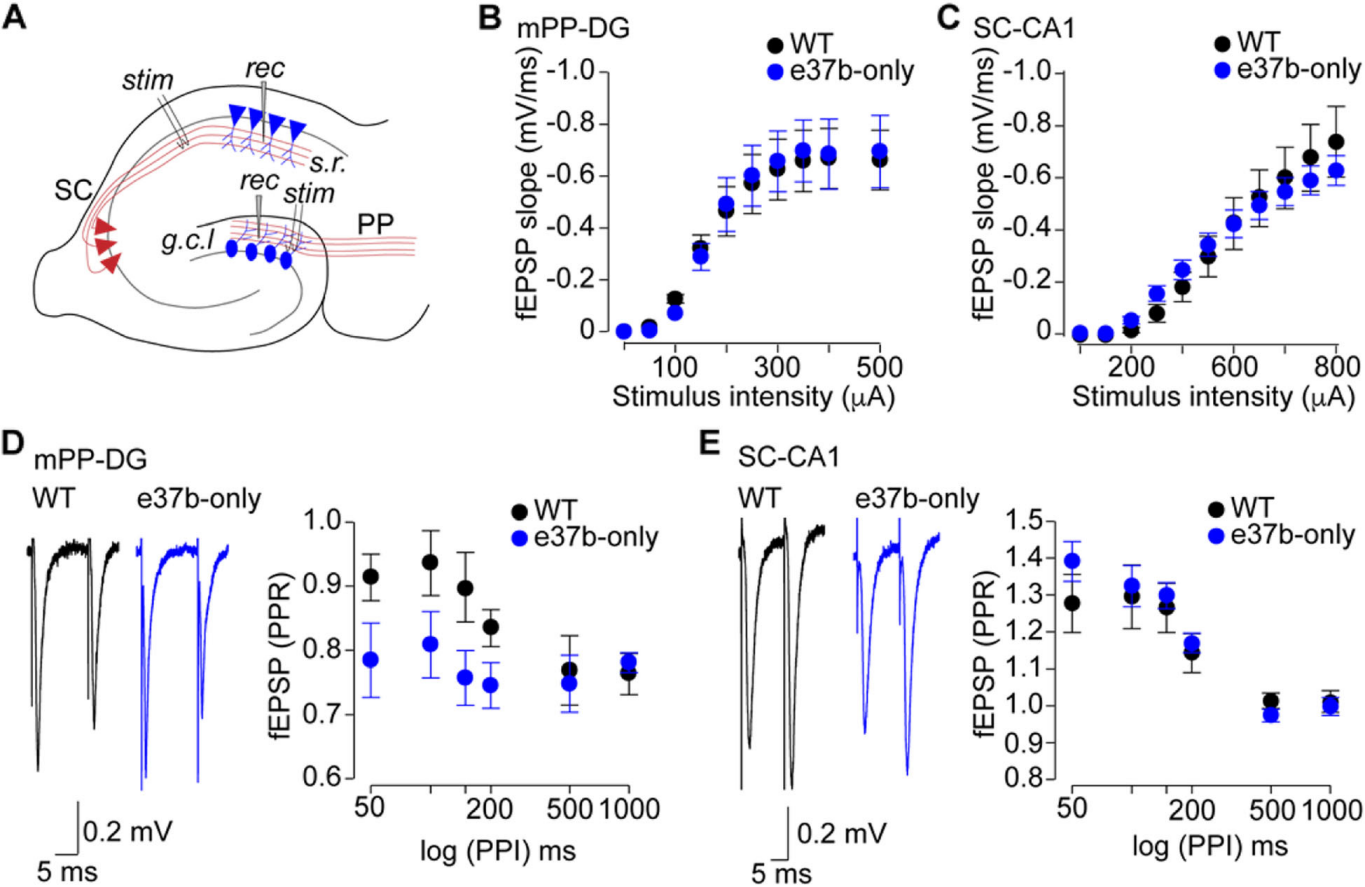
Replacing e37a-*Cacna1b* mRNA by *e37b-Cacna1b* results in enhanced probability of transmitter release in mPP-DG synapses. **a** Schematic describing approximate placement of recording (rec) and stimulation (stim) electrodes in the superior blade of DG and *s.r.* in CA1. **b** I/O relationships of fEPSPs from mPP-DG synapses recorded in WT and e37b-only mice. Slopes of fEPSPs were plotted as a function of the stimulus intensity. **c** I/O of fEPSPs from SC-CA1 synapses recorded in WT and e37b-only mice. Slopes of fEPSPs were plotted as a function of the stimulus intensity. **d** Representative traces of mPP-DG fEPSPs using a PPR protocol in WT and e37b-only slices (*left panel*). PPR of fEPSPs recorded at varying pulse intervals in slices of WT and e37b-only (*right panel*). **e** Representative traces of SC-CA1 fEPSPs using a PPR protocol in WT and e37b-only slices (*left panel*). PPR of fEPSPs recorded at varying pulse intervals in slices of WT and e37b-only mice (*right panel*). **b-e**. Data are shown as mean ± SE

Glutamate release at two key synapses of hippocampus, mPP-DG and Schaffer Collaterals-CA1 (SC-CA1) depend, in part, on Ca_V_2.2 channel activity [39]. Therefore, we compared synaptic transmission at mPP-DG and SC-CA1 synapses in vHPC of WT and e37b-only mice (Fig. 4a). We recorded fEPSPs by stimulating mPP and SC and recording in DG and CA1, respectively (Fig. 4a). fEPSPs at mPP-DG and SC-CA1 synapses were not obviously different between WT and e37b-only mice based on fEPSP input/output relationships (I/O). The relationship between fEPSP slope and electrical stimulation intensity were similar between WT and e37b-only mice for both synapses (F_1, 13_ = 2.4, *p* = 0.941, repeated-measures ANOVA, WT: *n* = 8; e37b-only: *n* = 7. Fig. 4b) and (F_1, 13_ = 4.9, *p* = 0.85, repeated-measures ANOVA, WT: *n* = 8; e37b-only: *n* = 7. Fig. 4c). We have previously shown that Ca_V_2.2 protein levels are similar between WT and 37b-only mice in the brain ([19], supplementary Figure 3). Those results combined with our synaptic physiology suggest that the absence of 37a-*Cacna1b* mRNA does not affect basic synaptic function of mPP-DG and SC-CA1 pathways. To determine if e37a-Ca_V_2.2 channels have a presynaptic role, we performed paired-pulse ratio (PPR) at these synapses as described below.

Presynaptic Ca_V_2.2 channels influence short-term plasticity [40, 41], which is a property linked to presynaptic calcium entry. Given that e37a influences both the number of Ca_V_2.2 channels trafficked to the cell surface as well as GPCR inhibition of Cav2.2 channel function [19, 28], we analyzed short-term plasticity using PPR at mPP-DG and SC-CA1 synapses of WT and e37b-only mice. We used a stimulus intensity adjusted to 30–40% of maximum for each individual recording (Fig. 4b, c). At mPP-DG synapses, we observed a consistent difference in the size of paired-pulse inhibition at short intervals (50–200 ms) between WT and e37b-only recordings (F_1, 13_ = 0.75, *p* = 0.01, repeated-measures ANOVA, WT: *n* = 8, e37b-only: *n* = 7). The average PPR at wild-type synapses was 0.9 compared to 0.7 at synapses of e37b-only mice. Thus, our data suggest that at short intervals, transmitter release probability is higher at mPP-DG synapses of e37b-only mice compared to WT (e37b plus e37a). At longer stimulus intervals, (0.5–1 s), there was no consistent difference in the size of paired pulse inhibition between WT and e37b-only recordings (F_1, 13_ = 1.5, *p* = 0.9, repeated-measures ANOVA, WT: *n* = 8, e37b-only: *n* = 7). SC-CA1 synapses facilitate in response to paired stimuli applied at intervals shorter than 200 ms. There were small, but inconsistent differences in paired pulse facilitation of fEPSP at SC-CA1 synapses from WT and e37b-only mice (F_1, 13_ = 1.98, *p* = 0.84, repeated-measures ANOVA, WT: *n* = 8, e37b-only: *n* = 7) and at longer intervals where there was no facilitation, thus PPRs were not consistently different in WT and e37b-only recordings (F_1, 13_ = 1.03, *p* = 0.92, repeated-measures ANOVA, WT: *n* = 8, e37b-only: *n* = 7).

Our findings suggest that mPP-DG synapses containing e37a-Ca_V_2.2 channels as compared to all e37b-Ca_V_2.2 containing synapses, have reduced release probability. This influences the size of the synaptic response when stimuli occur in rapid succession (intervals 200 ms or less). This result might be consistent with enhanced inhibition of e37a-Ca_V_2.2 channels by GPCRs compared to e37b-Ca_V_2.2 channels at mPP termini, as observed in studies of e37a and e37b clones expressed in cell lines [19, 28, 29]. By contrast, either e37a-Ca_V_2.2 channels are not expressed at SC termini or their unique properties do not influence synaptic transmission at SC-CA1 synapses based of the experiments performed in our studies. We next analyzed the subcellular distribution of e37a-*Cacna1b* mRNAs in EC which contains cell bodies of mPP axons that project to DG [42–44].

### e37a-Cacna1b mRNAs are enriched in CaMKIIα expressing projection neurons

**Fig. 5:**
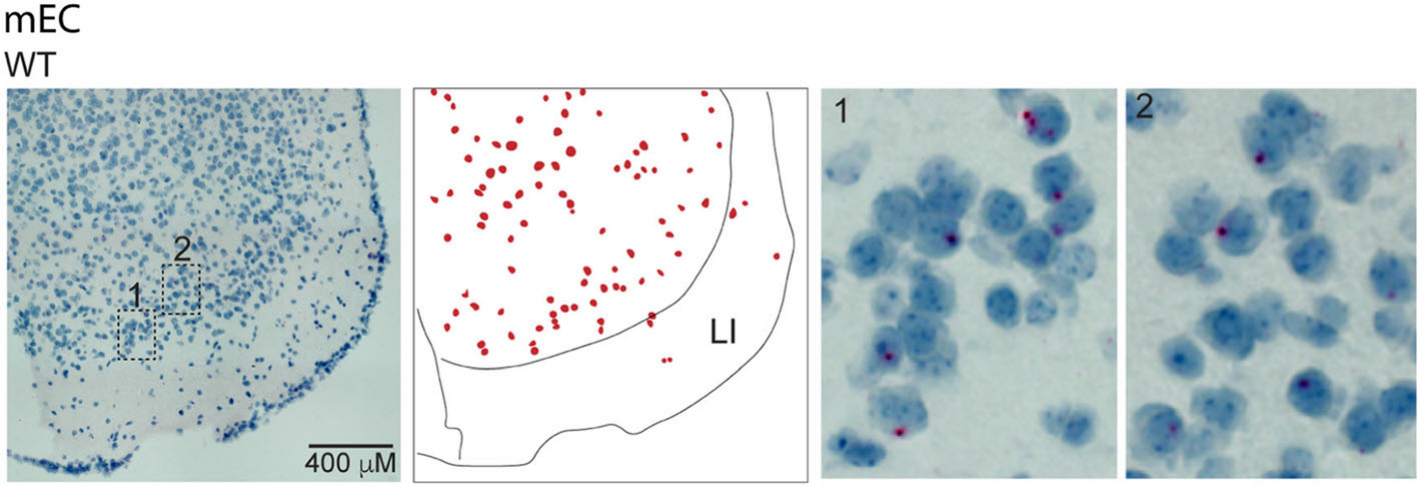
Localization of e37a-*Cacna1b* mRNA medial in entorhinal cortex. Representative BaseScope™ images of sections of mEC from WT and e37b-only mice. *Middle panels* show approximate localization of cell bodies stained for e37a-*Cacna1b*, this image was constructed based on *right panel* image. Insets 1 and 2 from regions of mEC were amplified for clarity

**Fig. 6:**
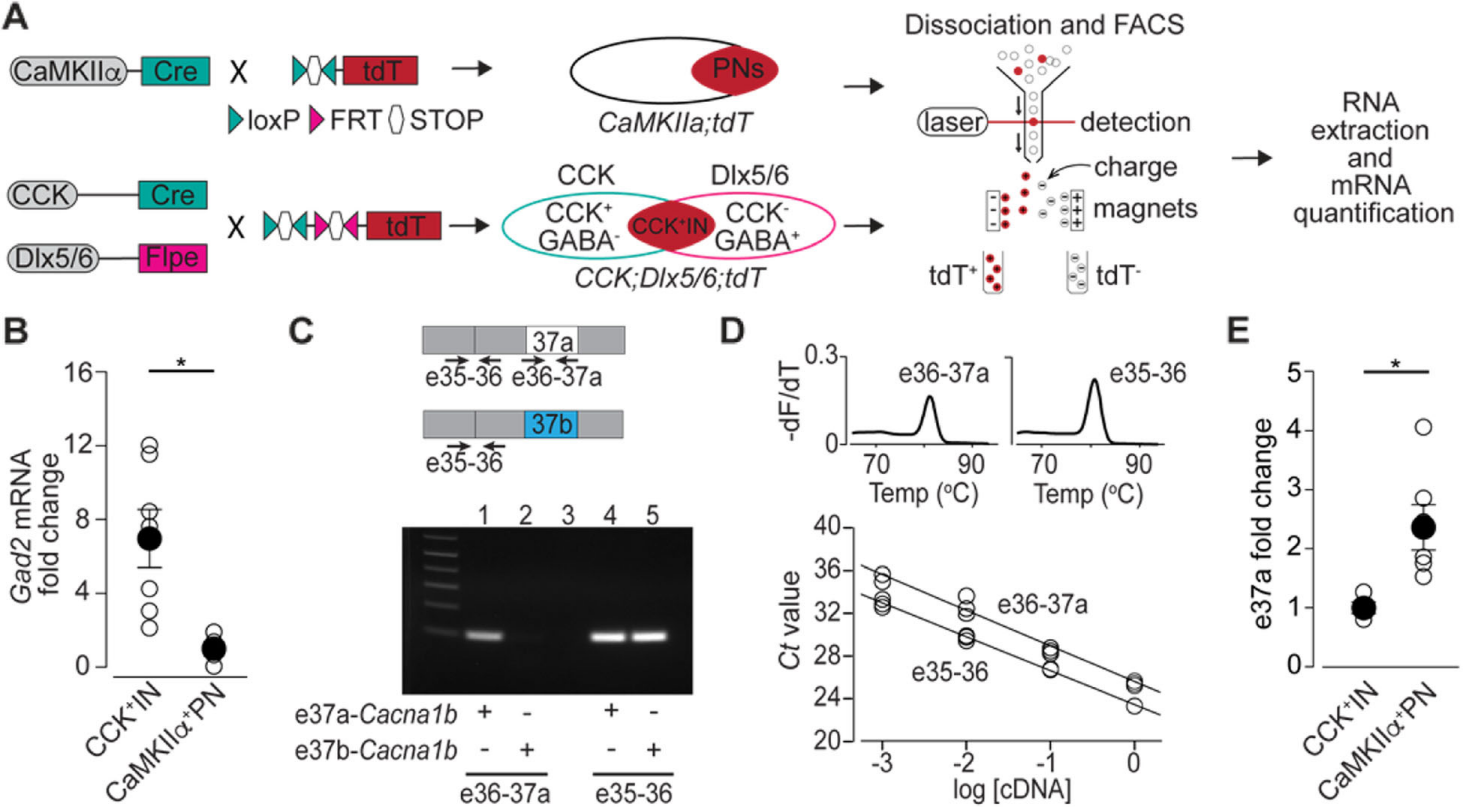
E37a-*Cacna1b* mRNA is more enriched in CaMKIIα^+^PNs relative to CCK^+^INs. **a** Schematic depicting the work flow to quantify 37a-Ca_V_2.2 mRNA in CamKIIα^+^PNs and CCK^+^INs, this includes genetic labeling using Cre/loxP and Flpe/FRT systems, neuronal dissociation, FACS and RT-qPCR. **b** Quantification of *Gad2* mRNA in total RNA isolated from CaMKIIα^+^PNs and CCK^+^INs. Data are shown as mean ± SE of *Gad2* fold change. *Gad2* mRNA expression was normalized to *Gapdh* mRNA levels. **c** *Upper panel*. Approximate location of primers (arrows) to amplify a sequence spanning e35–36 and e36-37a. *Lower panel*. Representative image to show the specificity of both sets of primers. Note the lack of amplification for e36-37a set of primers in the presence of the e37b-*Cacna1b* clone. As expected, e35–36 primers amplified both e37a*-Cacna1b* and e37b-*Cacna1b* clones. **d** *Upper panel*. Melting curve for e36-37a and e35–36 sets of primers. The derivative of fluorescence as a function of temperature was plotted (−dF/dT) against temperature. One single peak in each plot strongly suggest the presence of one product of amplification for both e36-37a and e35–36 sets of primers. *Lower panel*. Standard curves to assess the PCR efficiency for e36-37a and e35–36 sets of primers. Open circles indicate individual measurements of Ct values at a given dilution. All points for each sets of primers were considered to calculate slope of standard curve and PCR efficiency. **e** Quantification of e37a-*Cacna1b* mRNA in RNA isolated from CaMKIIα^+^PNs and CCK^+^INs. Data are shown as mean (filled symbols) ± SE, and individual values for each mouse (empty symbols). * *p* < 0.05

First, we probed for e37a-*Cacna1b* mRNAs using BaseScope™ in sections of EC. We found low levels of e37a-*Cacna1b* expression across the multiple layers of EC with the exception of LI (Fig. 5). To determine which cells express e37a-*Cacna1b* mRNA, we used a combination of genetic labeling and FACS coupled with RT-qPCR (Fig. 6a). All three Ca_V_2 channels (Ca_V_2.1, Ca_V_2.2, and Ca_V_2.3) contribute to synaptic transmission at excitatory SC-CA1 and mPP-DG synapses [45–47]. By contrast, synaptic transmission at CCK^+^INs termini which synapse onto granular cells of DG depend exclusively on the activity of Ca_V_2.2 channels [48–50]. We compared e37a-*Cacna1b* mRNA expression in PNs and CCK^+^INs using mice expressing the red fluorescent protein, tdT, in CaMKIIα-expressing cells to mark PNs (CaMKIIα^+^PNs) [51] and in CCK- expressing cells to mark CCK^+^INs (Fig. 6a). Figure 6a shows a schematic of the genetic labeling for CaMKIIα^+^PNs and intersectional labeling of CCK^+^INs (and see methods). We used the interneuron marker, *Gad2* to demonstrate enrichment of this mRNA in CCK^+^INs relative to CaMKIIα^+^PNs (Fold change, mean ± SE. CCK^+^IN = 6.96 ± 1.56, *n* = 7; CaMKIIα^+^ PN = 1 ± 0.41, *n* = 5. Mann-Whitney U test, *p* = 0.012, Fig. 6b).

To quantify e37a-*Cacna1b* mRNAs in CaMKIIα^+^PNs and CCK^+^INs, we used two sets of primers to amplify between e36 and e37a, and a second pair between e35 and e36 (e35–36) (Fig. 6c and see methods). The specificity of e37a primers was confirmed using plasmid cDNAs containing either e37a- or e37b-*Cacna1b* (Fig. 6c, lanes 1 and 2, respectively). The PCR pair in constitutive exons e35 and e36 amplified in both e37a and e37b-*Cacna1b* cDNAs (Fig. 6c, lane 4 and 5). Melting curve analyses show that both primer pairs amplify a single product (Fig. 6d, *upper panels*), and Ct values at serial dilutions of cDNA derived from DRG show that both primer pairs have similar efficiencies (e36-37a = 105 ± 3%, *n* = 3 and e35–36 = 106 ± 3%, *n* = 3. Fig. 6d, *lower panel*).

By normalizing to e35-e36 levels, we found that e37a-*Cacna1b* mRNA levels were consistently higher in CaMKIIα^+^PNs compared to CCK^+^INs (Fold change, mean ± SE. CCK^+^INs = 1.02 ± 0.098, *n* = 4. CaMKIIα^+^ PNs = 2.40 ± 0.385, *n* = 7. Mann-Whitney U test, *p* = 0.02. Fig. 6e). Therefore, e37a-*Cacna1b* mRNAs are expressed in projection neurons some of which form mPP-DG synapses.

### E37a-Ca_V_2.2 influences behavioral responses to aversive stimuli in mice

**Fig. 7:**
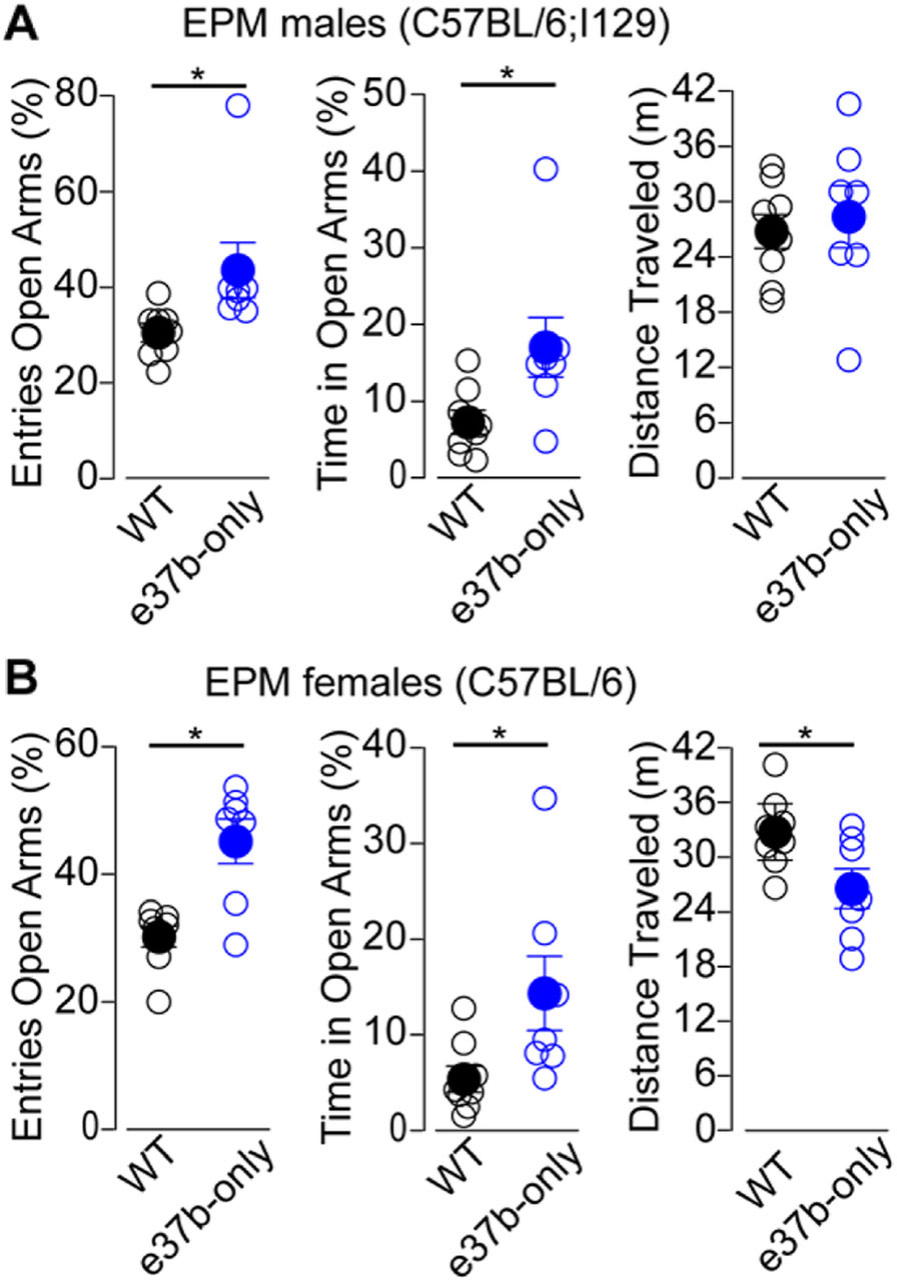
E37a-*Cacna1b* suppresses exploratory behavior in EPM test in males and females. **a** and **b** % of entries into OA, *left panel*. % of time spent in OA, *middle panel*. Total distance traveled, *right panel*. Data are shown as mean (filled symbols) ± SE and individual values (empty symbols). * *p* < 0.05

To determine if the presence of e37a-Ca_V_2.2 channels are important for certain aspects of mouse behavior, we compared WT and e37b-only mice in a series of exploratory and novelty-induced anxiety-like behaviors. E37b-only mice were compared to WT mice in elevated plus maze (EPM) and novelty-induced hypophagia (NIH) assays. We first tested WT and e37b-only male mice in C57BL/6;I-129 background. In EPM, e37b-only mice entered more frequently and spent more time in open arms (OA) relative to WT (% entries in OA, mean ± SE: WT = 30.8 ± 1.7%, *n* = 8; e37b-only, 43.7 ± 5.7%, *n* = 7. Mann-Whitney U test, *p* = 0.004. Fig 7a, *left panel*. % time spent in OA, mean ± SE: WT = 7.2 ± 1.6, *n* = 8; e37b-only = 17.0 ± 3.9, *n* = 7. Mann-Whitney U test, *p* = 0.021. Fig. 7a, *middle panel*). By comparison, no difference was detected in overall distance travelled in the elevated plus maze between WT and e37b-only mice (Distance traveled, mean ± SE: WT = 28.4 ± 3.4 m, *n* = 8; e37b-only = 26.7 ± 1.8 m, *n* = 7. Mann-Whitney U test, *p* = 0.49. Fig. 7a, *right panel*). Similar results were observed in female mice in C57BL/6 background (% entries in OA, mean ± SE: WT = 30.2 ± 1.6, *n* = 8; e37b-only = 45.2 ± 3.5, *n* = 7. Mann-Whitney U test, *p* = 0.011. Fig. 7a, *left panel*. % time spent in OA, mean ± SE: WT = 5.3 ± 1.4, *n* = 8; e37b-only = 14.3 ± 3.9, *n* = 7. Mann-Whitney U test, *p* = 0.021. Fig. 7a, *middle panel*). Interestingly, e37b-only female mice showed reduced locomotor activity compared to WT female mice (Distance traveled, mean ± SE: WT = 32.74 ± 3.09 m, *n* = 8; e37b-only = 26.53 ± 2.14 m, *n* = 7. Mann-Whitney U test, *p* = 0.041. Fig. 7a, *right panel*). Our results suggest that e37a-Ca_V_2.2 channels have an inhibitory influence, relative to e37b-Ca_V_2.2 channels, on exploratory behavior in elevated plus maze in both males and females. Furthermore, we also show that e37a-Ca_V_2.2 channels enhance overall locomotion in female mice, but not male mice compared to e37b-Ca_V_2.2 channels.

**Fig. 8:**
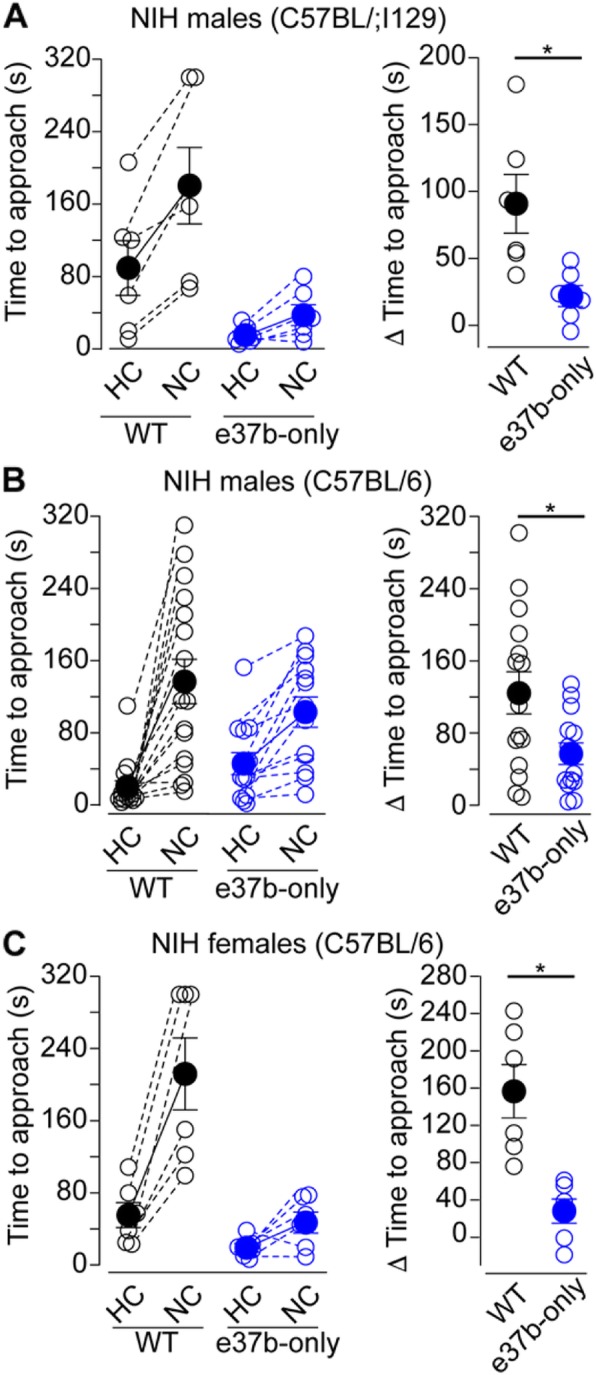
e37a-*Cacna1b* enhances behavioral responses to aversive stimuli. **a-c** *Left panel*, time to approach to reward in home cage (HC) and novel cage (NC) for male mice in C57BL/6;I129 (**a**), male mice in C57BL/6 (**b**) and female mice in C57BL/6 (**c**) for both genotypes. Dotted lines link time to approach from the same individual in HC and NC conditions for both genotypes. *Right panel*, difference in time to approach between HC and NC. All data are shown as mean (filled symbols) ± SE and individual values (empty symbols). * *p* < 0.05

Next, we assessed the ability of mice from both genotypes to resolve a conflict between a context that induces heightened aversion and a drive to approach an appetitive stimulus in the NIH assay (novelty-induced anxiety-like behavior). In this task, over the course of 3 days, rodents learn that a sipper tube delivers sweetened condensed milk. On the fourth day, latency to approach and drink in the home cage (HC) is measured. On the fifth day, mice are placed in a novel cage with a (NC) mildly aversive environment, and latency to drink is assessed. Mice typically show increases in latency to approach and drink from the sipper tube in this novel aversive environment, a marker of novelty induced anxiety-like behavior [52]. To determine the levels of novelty-induced anxiety-like behavior, time to approach in HC was subtracted from time to approach in NC (Δ Time to approach). E37b-only male mice in C57BL/6:I129 background overcame the aversive environment faster than WT mice from similar background (Δ Time to approach, mean ± SE: WT = 90.9 ± 20.3 s, *n* = 7; e37b-only, 20.4 ± 7.7 s, *n* = 7. Mann-Whitney U test, *p* = 0.01. Fig. 8a, *right panel*). We confirmed this result in e37b-only and WT mice in C57BL/6 (Δ Time to approach, mean ± SE: WT = 124.3 ± 23.0 s, *n* = 15; e37b-only = 57.2 ± 11.9 s, *n* = 14. Mann-Whitney U test, *p* = 0.036. Fig. 8b, *right panel*). Similarly, latency to approach was shorter for e37b-only female mice compared to WT female mice in C57BL/6 (Time to approach, mean ± SE s: WT = 156 ± 29 s, *n* = 6; e37b-only = 53 ± 30 s, *n* = 7. Mann-Whitney U test, *p* = 0.016. Fig. 8b). Our results suggest that e37b-only mice are more likely to approach the reward than WT mice despite the aversive environment.

## Discussion

### Cell-specific expression of e37a-*Cacna1b* pre-mRNA in brain

It was previously shown that e37a-*Cacna1b* mRNA is more abundant in DRG than in brain; ~ 6% to ~ 1.2% of the *Cacna1b* splice isoforms contain e37a in DRG and brain respectively [27]. However, alternative splicing within cell populations of the brain varies substantially [53]. Here, we show that ~ 8.5% of cells in cortical areas contain e37a, and these cells are distributed throughout the cortex. It is important to note that approximately ~ 1.7% of cells in sections from e37b-only mice showed signal for e37a. However, a combination of BaseScope™, FACS of genetically labeled neuronal subpopulations coupled to RT-PCR and e37b-only mice demonstrate that e37a-*Cacna1b* mRNAs are expressed more abundantly in CaMKIIα^+^PNs relative to CCK^+^INs.

### Alternative splicing in *Cacna1b* pre-mRNA and control of transmitter release

Interestingly, the other two members for the Ca_V_2 family, *Cacna1a* (Ca_V_2.1) and *Cacna1e* (Ca_V_2.3) also contain alternatively spliced exons that are homologous to e37a and e37b in *Cacna1b* [25, 54]. Recently, Thalhammer et al. demonstrated that switching e37a to e37b splice variants in *Cacna1a* resulted in reduced probability of transmitter release in excitatory synapses. This was attributed to differential coupling of e37 *Cacna1a* splice variants to the neurotransmitter release machinery [55]. Given that e37b-only mice in our study show reduced PPR compared to WT mice, our results suggest that switching splicing from e37a to e37b produced an increase in probability of transmitter release. Our findings oppose to those ones for 37 *Cacna1a* splice variants. The following can help to reconcile these apparently contradictory findings. e37a in *Cacna1b* is thought to enhance inhibition of calcium entry through Ca_V_2.2 channels by G_i/o_PCRs [19, 28]; therefore, by eliminating e37a-Ca_V_2.2 channels there might be a reduction in the inhibition of presynaptic calcium entry by G_i/o_PCRs, and therefore increased probability of transmitter release. Nonetheless, all these findings open the door for future exciting research on the role of splice variants of Ca_V_2 channels in neurotransmission.

### *Cacna1b* pre-mRNA alternative splicing and behavior

Here we show that although e37a-*Cacna1b* is expressed at very low levels in the brain [27], it has robust effects on the response to aversive stimuli in both males and females and on overall locomotion in female mice. The behavioral effects are not related to changes in overall protein levels because the amount of Ca_V_2.2 is similar in whole brain of both 37b-only and WT mice ([19], supplementary Figure 3). Developmental compensation by other Ca_V_s is unlikely to explain our behavioral observations, because the protein levels of Ca_V_2.1 channels is similar in whole brain of 37b-only and WT mice ([19], supplementary Figure 3). Furthermore, the non-N-type current recorded in DRG in both new born and adult mice is similar between 37b-only and WT mice, which provides more support to the lack of developmental compensation by other Ca_V_s in our genetic mouse models [19, 31]. All these observations are also in line with our electrophysiological results, where we were unable to observe differences in I/O relationships in excitatory synapses of vHPC.

The mechanisms underlying the behavioral effects of disrupting e37a splicing are yet to be determined. Our studies in hippocampus suggest a link between *Cacna1b* alternative splicing, hippocampal function, and behavioral responses to aversive stimuli. This link is supported by previous studies where direct excitation of DG increases exploratory behavior in rodents in the elevated plus maze [56, 57]. However, a limitation of our study is that we are unable to conclusively determine what areas of the nervous system are affected by disruption of e37a splicing in *Cacna1b* to influence exploratory behavior, given that e37a-*Cacna1b* is broadly expressed in CaMKIIα^+^PNs and CCK^+^INs, and possibly other types of interneurons. Nonetheless, our study is one of the very few linking single splicing events in *Cacna1b* to complex behavior.

## Data Availability

All data generated and analyzed during this study are included in this article. All our mouse lines are available at MMRRC repository or Jackson laboratories.
